# PI3K/PTEN/AKT Signaling Pathways in Germ Cell Development and Their Involvement in Germ Cell Tumors and Ovarian Dysfunctions

**DOI:** 10.3390/ijms22189838

**Published:** 2021-09-11

**Authors:** Massimo De Felici, Francesca Gioia Klinger

**Affiliations:** Department of Biomedicine and Prevention, University of Rome Tor Vergata, 00133 Rome, Italy

**Keywords:** primordial germ cells, PI3K/PTEN/AKT, ovarian follicles, ovarian reserve, primordial follicles, ovarian dysfunction, germ cell tumors

## Abstract

Several studies indicate that the PI3K/PTEN/AKT signaling pathways are critical regulators of ovarian function including the formation of the germ cell precursors, termed primordial germ cells, and the follicular pool maintenance. This article reviews the current state of knowledge of the functional role of the PI3K/PTEN/AKT pathways during primordial germ cell development and the dynamics of the ovarian primordial follicle reserve and how dysregulation of these signaling pathways may contribute to the development of some types of germ cell tumors and ovarian dysfunctions.

## 1. Introduction

Primordial germ cells (PGCs) are embryonic germ cell precursors. They derive from a subset of epiblast cells that migrate to the extraembryonic region shortly before gastrulation, prior to reentering into the embryo proper and moving into the gonadal ridges (GRs). Here, they give rise to oocytes or prospermermatogonia (the fetal precursors of spermatogonia) in the developing fetal ovaries and testes, respectively. This sequence of events occurs in all mammalian species studied including humans, although the involved factors and timing can be different. Nevertheless, from the experimental evidence accumulated during the last thirty years, a correct activation level and regulation of the PI3K/PTEN/AKT signaling pathways are conserved features of the mammalian PGC development. 

In the first part of the present paper, we review the main data obtained by several laboratories including our own, supporting this notion mainly with regard to PGC survival/proliferation and migration. Later on, in the establishing of the follicle stockpile in the ovary and its dynamics, the PI3K/PTEN/AKT signaling pathways continue to exert a pivotal role in regulating the activation of the primordial follicles and the oocyte growth. The second part of this work is focused on these subjects. 

In both sections, we report evidence on how dysregulation of the PI3K/PTEN/AKT signaling pathways may contribute to development of some types of germ cell tumors arising from PGCs or ovarian dysfunctions involving the follicle dynamics control.

Readers interested in these topics can also refer to the optimal reviews by Makker-vet and colleagues [1] and Maidarti and colleagues [2].

## 2. Brief Outline of PI3K/PTEN/AKT Signaling Pathways

**Figure 1:**
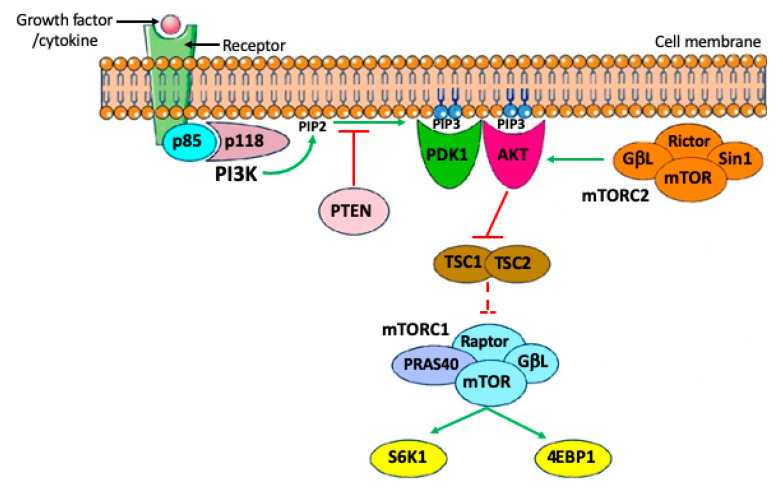
Simplified PI3K/PTEN/AKT signaling pathways discussed in the present paper.

Phosphoinositide 3-kinases (PI3Ks) are lipid kinases that play a central role in the regulation of a variety of cell activities including apoptosis, autophagia, cell cycle, differentiation, and motility. They are activated downstream tyrosine kinase receptors (RTKs) and/or G proteins coupled receptors (GPCRs). Four major extracellular signals, growth factors, cytokines, hormones/ chemokines, and integrins, are able to activate PI3Ks. In the cytoplasm, PI3K phosphorylates the 3′-hydroxy position of the inositol ring of phosphatidylinositides, yielding to the production of phosphatidylinositol-3,4,5-trisphosphate (PIP3), a second messenger that recruits protein kinase B (AKT) to the cell membrane by phosphorylation. This step is tightly regulated by the phosphatase and tensin homolog (PTEN), which acts as a negative regulator of the pathways by converting PIP3 back to PIP2. To be fully active, AKT requires an additional phosphorylation by mTORC2, after which it is able to phosphorylate several substrates. To date, at least 13 substrates of AKT have been identified in mammalian cells [3]. These substrates have been classified into two major groups: apoptosis regulators and cell growth regulators. One of the most important pathways downstream of AKT is the mammalian target of rapamycin (mTOR). mTOR is a major regulator of cell growth and metabolic state in response to nutrients, growth factors, and many extracellular signals. mTOR assembles into two functionally distinct complexes: mTOR complex 1 (mTORC1) and mTOR complex 2 (mTORC2). AKT signaling stimulates mTOR within mTORC1 to directly phosphorylate the ribosomal S6 kinases, S6K1 and S6K2, and the eIF4E-binding proteins, 4EBP1 and 4EBP2, in order to promote protein synthesis and ultimately cell survival, growth, and proliferation. In such a contest, mTORC1 activity is able to inhibit cell apoptosis and autophagy by various mechanisms (Figure 1).

## 3. The PI3K/PTEN/AKT Signaling Pathways in PGC Development

It is now largely accepted that in mammalian embryos, the germline is specified from a small cluster of cells of the epiblast, the layer of the blastocyst known to generate all fetal cell lineages, shortly before gastrulation. Under the inductive action of WNT and BMP growth factor signaling, which basically rely on the SMAD-and β-catenin pathways [4,5], these cells express transcription factors such as OCT4, SOX-2 or -17, NANOG, PRDM-1, and -14 that begin to reprogram their genome to a naïve pluripotent status and at the same time prevent their differentiation into somatic cell lineages [6,7]. These processes continue and are reinforced in the wall of the yolk sac, where the specified germ cells move. Here, a new round of induction occurs that predetermines these cells as the germ cell precursors termed primordial germ cells (PGCs). PGCs, destined to give rise to the gametes after colonization of the gonadal anlages within the embryo proper, are now characterized by the expression of surface markers such as the alkaline phosphatase enzyme (APase) and Stage Specific Embryonic Antigen (SSEA) oligosaccharides, typical of pluripotent stem cells, RNA-binding proteins such as TIAR, LIN28 and DND1, and Nanos3, important regulators of cell survival and self-renewal [8,9,10,11]. 

During this period, PGCs also express the KIT receptor for the Kit ligand (KL) cytokine and receptors for growth factors such as fibroblast growth factors (FGFs) or other cytokine such as leukemia inhibitory factors (LIF), the LIFR-gp180 complex [12,13,14]. These receptors have been localized on the membrane of migratory and post migratory PGCs and in vitro studies proved that their activation by the respective ligands, KL (or SCF), bFGF (or FGF2) and LIF or related cytokines, promotes PGC survival, migration, and/or proliferation [13,14,15,16,17]. However, the downstream effectors of the activation of these receptors remain poorly characterized. In fact, of the three intracellular signaling pathways known to be activated by LIFR-gp180: JAK (Janus kinase)/STAT3 (Signal transducer and activator of transcription 3), PI3K/AKT, and SHP2 [SH2 (Src homology 2) domain-containing tyrosine phosphatase 2]/MAPK (Mitogen-activated protein kinase), only this latter is probably activatable by LIF in PGCs [18]. As far as we know, of the three possible signaling pathways downstream FGFR activation, RAS/RAF/MAPK, PI3K/AKT, and phospholipase Cγ, the first are probably activatable by FGF in migratory and perhaps in post migratory PGCs [13]. Conversely, more information is available for KL/KIT system signaling. As a matter of fact, natural or induced mutants confirmed a crucial role for PGC survival and possibly migration in vivo only for the KL/KIT system [14,19].

### 3.1. KL/KIT in PGCs as a Major Effector of PI3K/AKT Signaling in PGCs

KL, known as KITL or stem cell factor (SCF), exists as a membrane-anchored and soluble isoform that arises from alternative RNA splicing and proteolytic processing [19,20]. It is obvious to postulate that the membrane-anchored KL participates in short-range signaling transmitted by cell–cell contacts whereas the soluble isoform takes part in longer-range signaling transmitted by diffusion through the extracellular medium. Both isoforms seem able to activate KIT in PGCs [21], but no information exists if they are able to activate different signaling pathways. 

KIT belongs to the type III receptor tyrosine kinase family, and it is well known that stimulation of KIT by KL leads to dimerization, activation of its intrinsic tyrosine kinase activity, and phosphorylation of key tyrosine residues within the receptor. The resulting phosphotyrosine residues serve as docking sites for molecules containing SH2 and other phosphotyrosine-binding domains. Activated KIT also catalyzes direct phosphorylation of substrate proteins. Among the major signaling pathways ultimately activated by the KL/KIT including RAS-RAF-MEK-ERK, PLCγ, Src, and PI3K/AKT, these two latter are probably the most important to sustain PGC survival and take part in their active migration toward the gonadal anlages [22,23,24]. Interestingly, we found that the binding of 17-β-estradiol to its receptor ERα was also able to induce a rapid PI3K-dependent AKT phosphorylation in mouse PGCs [25].

It can be postulated that in post migratory PGCs as in other cell types, the p85 regulatory subunit of PI3K binds to phosphotyrosine residues Y719 of activated KIT by one or both of its SH2 domains, and p85–p110 interaction leads to the allosteric activation of the catalytic subunit of the enzyme [26,27]. Upon PI3K activation, a cascade of events leads to the canonical AKT phosphorylation and activation described in the previous section. The fact, however, that induced mutations, which disrupt the Tyr719 binding site for the regulatory p85 subunit of PI3K in the KIT receptor [3,18], did not significantly affect the development of mouse PGCs, seems to contradict the notion about a crucial role of PI3K in PGC development. On the other hand, in vitro incubation of mouse PGC with KL results in rapid phosphorylation of AKT that is abolished by PI3K inhibitors [17,19]. Reconciling these apparently contrasting results, we demonstrated that in mouse PGCs, the KIT autophosphorylation activity efficiently stimulated by KL was associated with increased AKT phosphorylation mediated by either PI3K or Src kinases. Therefore, in the absence of phosphorylated Y719, it is likely that p85 is recruited by other signaling or scaffold proteins, for example, Grb2 or Src, that finally activate PI3K [20,21]. 

In the same paper, a novel role for KL as the PGC chemoattractant and for PI3K/AKT and Src kinase, as players involved in the activation of the PGC migratory machinery, were demonstrated. In addition, evidence for two other possibly PI3K-dependent KL/KIT action on PGCs, adhesion to somatic cells [22], and survival, this latter, due to a reduction in the expression of the pro-apoptotic Bax gene, have been previously reported [23,24]. Gu’s group [15] found that the membrane-bound form of KL maintained a high local concentration for mouse PGC motility and defined their region of migration.

**Figure 2:**
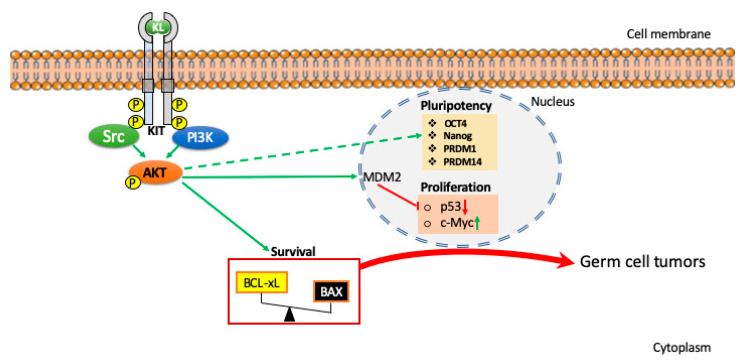
Schematic representation of KL/KIT-dependent PI3K/AKT signaling pathways identified in mouse PGCs involved in their survival/proliferation and possibly in their tumorigenic transformation as described in the text. The role of AKT in sustaining the expression of pluripotency transcription factors is represented as hypothetical (dotted line).

Among more 16 substrates downstream AKT, which ones might be directly or indirectly involved in PGC migration and survival are not known. Some of these are indicated in Figure 2 and discussed in De Felici and Klinger [25].

### 3.2. The PI3K/PTEN/AKT Signaling Pathways in the Generation of Germ Cell Tumors from PGCs 

Germ cell tumors (GCTs) represent neoplasms derived from germ cells that can contain both immature and mature elements differentiating into several tissue types. Gonads are the preferred location for the onset of these tumors; however, they can also be found in extragonadal sites (EGCTs). EGCTs are usually seen in children or young adults and typically arise in midline locations where the cranium and sacrococcygeal region are the most common sites. In testis, GCTs are termed testicular germ cell tumors (TGCTs); in ovaries, ovarian germ cell tumors (OGCTs). Histologically, EGCTs and TGCTs comprise seminomas (SEs), also known as germinoma, and nonseminomatous tumors (NSTs). The latter includes a variety of histological distinct types such as teratoma, embryonal carcinoma, endodermal sinus tumor (yolk sac tumor), choriocarcinoma, and tumors with mixed histology. Nonseminomas containing a pluripotent component known as embryonal carcinoma (EC) cells are known as teratocarcinomas. There are four main types of OGCTs, namely dysgerminomas, comparable to testicular seminomas, and teratoma, yolk sac tumor and choriocarcinoma histologically equivalent to their testicular counterparts. Further information and clinical outcomes of these tumors were not subject of the present review. We refer the interested readers to our recent reviews [28,29]. Here, we focus on their possible common origin from PGCs.

In fact, a widely accepted hypothesis suggests that EGCTs and the most part of TGCTs and OGCTs arise from PGCs with altered survival, deregulated cell cycle, and failed complete germline determination, probably acting together. A theory concerning the tumorigenic potential of extragonadal germ cells known as the ‘germ cell theory’ was first proposed by Gunnar Teilum, suggesting that EGCTs originate from stray migratory PGCs [26]. PGCs migrate across the embryo toward the developmental gonads, where they differentiate into gametes and require guidance cues to survive and reach their final destination. Moreover, after reaching the GRs, they are finally determined as germ cells and proliferate actively as oogonia before entering meiosis or prospermatogonia destined to undergo mitotic arrest in the differentiating ovaries and testes, respectively.

It is likely that the correct development of PGCs moving from the yolk sac toward the GRs will depend on the balance between the cytokine/growth factor signaling pathways that control their cell cycle and survival and the core transcription factors and RNA binding proteins that maintain their intrinsic pluripotency and preserve their germline identity by repressing differentiation toward somatic lineages. As a matter of fact, the latent pluripotency derived from specification and the highly hypomethylated status of the chromatin imposed to PGCs during migration represent a risk for tumorigenic transformation. 

As reported above, PGC migration is accompanied by a wave of KL expression by the surrounding somatic cells, which through KIT/PI3K/AKT pathways sustain their survival and facilitate migration [15]. Several papers have reported that enhanced activation of the PI3K/AKT pathways following ablation/inhibition of PTEN or continuous AKT hyperactivation delayed PGCs along their migratory pathway [22,25]. In the presence of KL, PGCs close to the GRs continue to migrate and colonize the gonads, while those remaining further away in the midline body structures normally die by the BAX-dependent apoptotic pathway in the absence of KL [15,27,28]. It has been suggested that this might be an elegant way to prevent aberrant PGC migration: PGCs that stray from their migratory pathway are eliminated through apoptosis [27]. However, if misplaced PGCs survive in extragonadal sites, they could, in certain microenvironments, remain undetermined and give rise to different types of tumors. In fact, over time, they might be stimulated to proliferate and, maintaining germ line specification, give rise to germinoma or, after losing the restriction to differentiate toward somatic lineages, generate nonseminomatous tumors. In fact, multiple lines of evidence suggest that PGCs are incompletely determined until they enter into the GRs, where they progressively lose pluripotency and somatic potential and acquire the definite germ cell fate. Similarly, after gonad colonization, dysregulation of PGC survival/proliferation associated with failed or partial determination as gametes is the likely cause of the formation of TGCTs and OGCTs. In this regard, pioneering experiments carried out in 129/Sv mice susceptible to spontaneously developing testicular teratomas have demonstrated that these tumors actually derived from PGCs [29], and that in these strains, PGCs within fetal testis continue to proliferate beyond the time they normally undergo mitotic arrest [30]. In supporting this notion, several evidence have subsequently indicated that most parts of the TGCTs, both in mouse and humans, originate from precursor cells termed germ cell neoplasia in situ (GCNIS), representing transformed PGCs or prospermatogonia [31]. 

The latent pluripotency of migratory and gonadal PGCs has been elegantly demonstrated by in vitro transformation of mouse or human PGCs in the presence of a cocktail of growth factors such as KL, LIF, and bFGF [32,33,34] to form pluripotent stem cells, known as embryonal germ (EG) cells, capable of forming teratomas when transplanted in the animal. 

One of the first mechanisms identified as responsible for PGC transformation in EG cells was the deregulation of cell cycle/survival signals linked to overactive PI3K/AKT pathway or downregulated PTEN [19,21,35], followed by upregulation of the transcription factors c-Myc, KLF4, and STAT3 [36,37]. At these stages, both KL and bFGF are likely to be responsible to activate the PI3K/AKT signaling pathways in PGCs. It has been elegantly demonstrated that in PGC-specific Pten-deficient mice, AKT was hyperphosphorylated and early teratomatous foci were also observed in fetal testes. In these mice, PGCs such as in the 129/Sv strains proliferated beyond the time they should undergo mitotic arrest and although the majority of them eventually die by apoptosis, a population survived to generate testicular teratomas [21]. Another target of AKT overexpression in transformed PGCs in vitro was identified in mouse double minute 2 homolog (MDM2), whose enhanced stability and nuclear localization suppressed p53 phosphorylation required for its activation [35] (Figure 2). Whether PI3K/AKT signaling pathways besides driving survival and proliferation play a role in inherently preserving the PGC pluripotent properties is not known, and should be investigated. Interestingly, PI3K inhibition in human embryonic stem cells (hESCs) resulted in the downregulation of pluripotency markers concomitant with the upregulation of lineage-specific genes, which together strongly suggest the loss of pluripotency (for a review and references here in, see [38]).

## 4. The PI3K/PTEN/AKT Signaling Pathways in Oogenesis and Folliculogenesis 

As reported above, upon colonization of the gonadal, female PGCs give rise to oogonia that after some rounds of mitotic proliferation enter meiosis I as primary oocytes. These are surrounded by ovarian pregranulosa cells and become progressively arrested at the diplotene/dictyate stage of prophase I and singularly enclosed in a structure termed primordial follicle (PMF). During the last period of prenatal development, cohorts of PMFs are recruited to begin growth. 

After birth, recruitment from the PMF pool continues until it has been depleted, at which point infertility ensues and the female is said to have entered reproductive senescence, or menopause in the case of humans [30]. 

Although recent evidence supports the possibility that in mammals, as occurs in other species, new follicles can be formed after birth [31,32], PMFs can remain quiescent within the ovary for a long time, even several decades in humans. The depletion of this stockpile occurs through atresia or recruitment into subsequent folliculogenesis stages. After recruitment, a process termed follicle activation, follicles only have two fates: to complete folliculogenesis by ovulation or undergo atresia (follicle death). Following activation, flattened pregranulosa cells surrounding the oocyte in PMFs differentiate into cuboidal cells and become mitotically active; continued proliferation drives the formation of multiple granulosa cell layers and the follicle transitions into first a primary, then a secondary or preantral follicle. Among the antral follicles, ovulatory follicles are finally selected. Only follicles able to adequately respond to FSH and LH gonadotropins survive and complete folliculogenesis with ovulation, whereas all others undergo atresia. 

Atresia of growing follicles seems to primarily begin in granulosa cells and subsequently encompass the oocyte. Granulosa cell apoptosis mostly depending on gonadotropins and steroids is widely considered as an underlying mechanism of follicular atresia. Conversely, atresia of PMFs is believed to accompany or follow oocyte death. 

In principle, as reported above, PMFs within an adult ovary should be subjected to mechanisms that keep them dormant, preserve survival, and at the same time, the oocyte quality, for long time. Autophagy and apoptosis, which appear to co-exist both in oocytes and granulosa cells, are likely to regulate oocyte survival and follicle atresia during folliculogenesis and PI3K/AKT signaling pathways to play a major role in either processes.

The notion that PI3K/AKT signaling pathways are major regulators of PF survival, mainly of the oocyte, but also of follicle activation as we will discuss later on, began to be uncovered in the early nineties. Similar to PGCs, the KL/KIT system in oocytes seems to be the major upstream activator of PI3K/AKT signaling pathways, although other growth factors and receptors, for example, IGF1/IGFR, are likely to contribute to the pathways [33,34]. The essential roles of KL/KIT in the oocyte and follicular development processes have been well-established using both in vitro and in vivo approaches including naturally occurred or induced mutations [35,36]. KIT is known to be present in oocytes at all stages of follicular development in postnatal ovaries of mouse, rat, and humans [37,38,39,40,41]. In contrast to KIT, KL is produced by the surrounding granulosa cells [38,41,42,43]. On the other hand, a variety of stimuli can regulate the PI3K/AKT signaling pathways in granulosa cells including different growth factors such as IGF-1 and EGF and physical and mechanical factors (for example, cell adhesion, ovarian tissue density, and Hippo pathways) [44,45,46,47,48,49]. A role of EGF in maintaining viability (but not promoting activation) of PFs by stimulating AKT phosphorylation has been demonstrated in prepubertal domestic cats [33]. Moreover, as we will briefly discuss in the last section, PI3K/AKT most likely has a role in the sophisticated mechanisms that allow the oocytes to repair DNA damage and contribute to preserve the oocyte quality.

From the above, it is easy to foresee that defects in PI3K/AKT signaling pathways can result in serious ovarian dysfunctions such as diminished ovarian reserve (DOR), premature ovarian insufficiency (POI), or premature ovarian failure (POF), and poor ovarian response (POR), characterized by reduced PMF/oocyte survival or defects in follicular activation. 

### 4.1. The PI3K/PTEN/AKT Signaling Pathways in Oocyte Survival/Apoptosis

**Figure 3:**
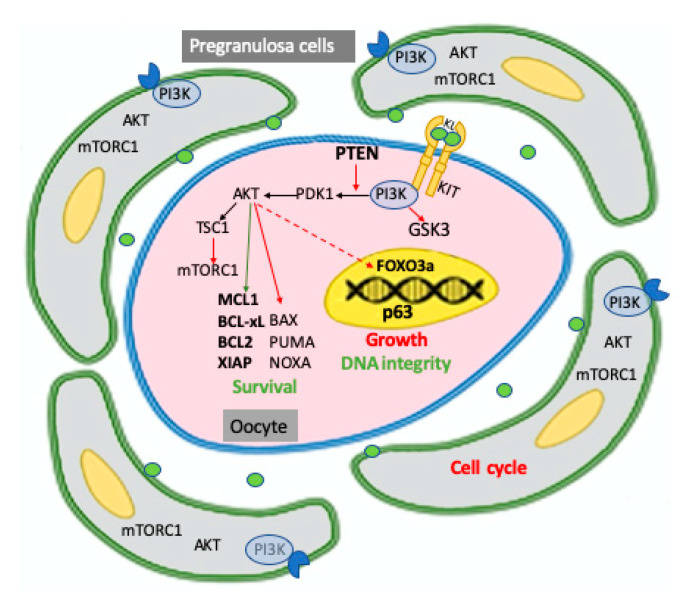
Schematic representation of PI3K/PTEN/AKT signaling pathways identified, mainly in the mouse, in the two components of the primordial follicle, pregranulosa cells, and oocyte, and of their major targets involved in maintaining the dormancy of the follicle including cell cycle in pregranulosa cells and survival, growth, and DNA integrity in the oocyte. As extensively discussed in the test, pregranulosa cells are arrested in the reversible GO cell cycle stage while the oocyte within the primordial follicle is maintained in a quiescent status, requiring the prevalence of survival upon proapoptotic factors, preservation of DNA integrity, and the inhibition of transcription necessary to its growth. In oocytes, all or a relevant part of these activities require the basal level of PI3K/AKT activity finely regulated by the KL/KIT system and PTEN. Activation or inhibition of AKT on its substrates are represented as green and red arrows, respectively; the inhibitory action of AKT on FOXO3a is represented with a dotted arrow since it is exerted only after oocyte activation. According to a current model, not illustrated in the figure, but reported in the test, signals from the microenvironment able to activate the PI3K/AKT/mTORC1 pathways in the pregranulosa cells reverse GO to the G1 stage and increment the production and release of factors, among these KL, which also incremented the PI3K/AKT/mTORC1 activity in the oocyte, leading to increased protein synthesis and removing the FOXO3a inhibition on its growth; in turn, the growing oocyte releases factors such as BMP15 and GDF9, which stimulate pregranulosa cell proliferation and their maturation into granulosa cells.

Summarizing and interpreting the numerous available data, it is possible to envisage that basal activation of PI3K/AKT signaling pathways are important components of the set of factors supporting the survival of oocytes within the PFs. The system likely acts by sustaining antiapoptotic pathways and perhaps maintaining the basal level of autophagy. However, the exact AKT substrates responsible for such actions have not been revealed thus far. Since the antiapoptotic myeloid cell leukaemia-1 (MCL1), to a lesser extent BCL-2 and BCL-xL and the proapoptotic BAX, PUMA and NOXA proteins, together with the initiator caspase 8 and the effector caspases 3 and 7, has been identified as major players of oocyte survival/apoptosis at this stage (for a review, see [50]), AKT activity could operate on the expression, localization, and/or activity of these proteins. Moreover, phosphorylation by AKT could stabilize endogenous apoptosis inhibitors, particularly X-linked inhibitor of apoptosis protein (XIAP), which inhibits the activity of the caspase 7/9, as suggested by Ene and colleagues and Klinger and colleagues [51,52]. At the same time, low AKT activity linked to that of mTOR, a well know effector of AKT, are compatible with basal autophagy under the condition of relative oocyte quiescence [53]. Interestingly, Sun et al. found that miR-378-3p maintains the size of the mouse PMF pool by increasing autophagy and inhibiting apoptosis by targeting PDK1, a master kinase crucial for AKT activation and caspase 9 transcripts, respectively [54] (Figure 3).

### 4.2. The PI3K/PTEN/AKT Signaling Pathways in Primordial Follicle Activation and Development

In the last few years, it has become evident that the PI3K/AKT activity downstream KL/KIT that sustains the prolonged survival of the oocyte is only part of the PI3K/AKT signaling pathways that play a major role in the maintenance of the PMF pool within the mammalian ovary including humans. In fact, these pathways are also involved in inducing the activation of dormant follicles, oocyte growth, and further follicular maturation [55,56]. It seems that loss of either PDK1 or PTEN, the two major regulators of the AKT activity downstream PI3K, can lead to premature ovarian failure (POF), but through different mechanisms. PTEN loss is associated with excessive PMF activation and subsequent follicular atresia, whereas PDK1 deficiency instigates accelerated clearance of PMFs straight from their quiescent state [57,58]. PTEN inhibition, as a central negative regulator of the pathway, has been widely used to activate PMFs in a range of species (for a review and references herein see [2].

Recent results indicate that the activity of mTOR downstream of the PI3K/AKT pathways in granulosa cells is a major trigger for PF activation [59]. Interestingly, Andrade’s group was able to identify in granulosa and cumulus cells from bovine growing follicles a molecular landscape formed by high PI3K-AKTactivity, low PTEN, FOXO3a, BAX levels, and high levels of miR-20a and mir-494 that downregulate PTEN levels, associated with high oocyte developmental potential [60]. This suggests that activation of the PI3K/AKT pathways in granulosa cells contribute to the acquisition of oocyte maturation during follicle development. Moreover, the activation of mTOR mediated by AKT suppresses rat granulosa cell autophagy during follicular atresia [61]. 

Robust evidence indicates that mTOR is an indirect effector of AKT in regulating oocyte growth and the subsequent follicular development [62,63]. AKT signals through phosphorylation of the TSC1/TSC2 complex to indirectly activate the mTORC1 complex. A crucial effector of mTORC1 is 40S ribosomal protein S6 kinase (S6K), which directly regulates ribosome biogenesis, cell cycle progression, protein synthesis, and metabolism. The increased protein synthesis in the oocyte includes secreted factors such as BMP15 and GDF9, which stimulate pregranulosa cell proliferation and sustains a molecular dialogue between the oocyte and the companion granulosa cells that continue during the rest of folliculogenesis.

Follicular activation also requires inactivation and removal from the oocyte nucleus of the transcription factor FOXO3a and the cyclin dependent kinase (CDK) inhibitor 1B (commonly known as p27kip1 or p27); this latter is also inactivated in pre-granulosa cells [64,65]. FOXO3a is a substrate of AKT, and is known as a transcription factor that leads to apoptosis and cell cycle arrest in many cell types. As a matter of fact, FOXO3a phosphorylated by AKT is excluded from the oocyte nucleus and is suppressed from functioning as a transcription factor, preventing its growth. Remarkably, overexpression of constitutively active FOXO3 in the nucleus of mouse oocytes preserves them in a dormant state [66]. FOXO3a inactivation by AKT could also have consequences for oocyte apoptosis and capability to repair DNA damage and counteract reactive oxygen species (ROS) effects that FOXO3a usually promotes (see below and [67,68,69]). In this regard, it has been recently reported that signaling by SIRT1, a NAD-dependent deacetylase that removes acetyl groups from various proteins including histones, acts through FOXO3a-MnSOD to protect mouse oocytes of growing follicles from oxidative stress [70]. Moreover, inactivation of FOXO3a downstream KL/KIT-dependent PI3K/AKT activity partly inhibited apoptosis occurring in naked rat oocytes isolated from PF [69]. If and how FOXO3a exerts these actions in the oocyte within the dormant PF, however, is not known. 

Finally, Liu and colleagues [71] reported that the PI3K/AKT activity downstream of KL/KIT led to phosphorylation/inactivation of GSK-3β and GSK-3β in cultured growing mouse oocytes, indicating that GSK-3 may participate in the regulation of mammalian oocyte growth and early follicular development. However, the molecules downstream of GSK-3 in mouse oocytes remain to be identified.

For the sake of truth, we must cite that although all results reported above strongly indicate a central role of KL/KIT in PMF activation, by employing a knock-in mutation (KitY719F) that completely abrogates signaling via PI3K, John’s group [72] observed that KIT had a specific role in the maintenance of PMF reserve and in the transition of primary to secondary follicles, but was dispensable in PMF activation in mice.

The importance of the mTOR/AKT signaling pathway for female fertility was, however, recently demonstrated in women suffering from POI/POF who, after receiving an AKT activator and the activation of Hippo signaling, were shown to achieve follicular maturation, IVF of oocytes, and healthy offspring (reviewed in [73]). 

### 4.3. The PI3K/PTEN/AKT Signaling Pathways in the Oocyte DNA Damage Response 

Under stress conditions, the apoptosis process of oocytes within PMFs is mediated by a distinct cell surveillance mechanism involving N-terminal transactivation domain p63 (TAp63α) [74,75], a p53 family member. Oocytes in the quiescent state demonstrate a high TAp63α expression and TAp63α functions primarily to respond to DNA damage caused by various agents including radiation, chemotherapeutic drugs, and likely ROS. As a matter of fact, TAp63α is thought to be the guardian of the oocyte genome during a long lifespan. Mouse oocytes within PFs also express all necessary kinases required to trigger TAp63 activation centered in a consecutive interplay of CHK2 and CK1 [76]. Transcriptional activation of PUMA and NOXA are critical downstream targets of oocyte apoptosis mediated by TAp63 [77]. As the other two members of the family p53 and p73, p63 can activate crucial genes involved in DNA repair such as, for example, Rad51, Brca2 and Mre1 [78], however, whether TAp63 in oocytes possesses such capability is not known. Similarly, it is not known whether in oocytes AKT is able to modulate TAp63 activation and its consequent transcriptional activities or conversely whether TAp63 can modulate AKT. In principle, this can be possible since it is well-known that AKT exerts negative control of p53 levels through enhancing MDM2-mediated targeting of p53 for degradation and that conversely, PTEN promotes p53 translation and protein stability [79]. Moreover, it has been reported that p63 plays a pivotal role in the progression of esophageal squamous carcinoma cells through regulation of the cell cycle via the Akt signaling pathway [80]. 

Remarkably, several recent studies have also implicated AKT in modulating DNA damage responses and genome stability (for reviews, see [2,81]). High AKT activity can suppress ATR/CHK1 signaling and homologous recombination repair (HRR) via direct phosphorylation of CHK1 or TopBP1 or, indirectly by inhibiting recruitment of double-strand break (DSB) resection factors such as RPA, BRCA1, and RAD51 to sites of damage. Conversely, AKT is activated by DNA double-strand breaks (DSBs) in a DNA-PK- or ATM/ATR-dependent manner and in some circumstances, can contribute to radio-resistance by stimulating DNA repair by nonhomologous end joining (NHEJ). In a recent paper [82], we found that KL added to the medium of cultured tissue fragments of early postnatal mouse ovaries partly protected oocytes of PMFs from apoptosis induced by cisplatinum, but did not prevent or favor DNA damage caused by the drug. However, whether AKT modulates the response of the oocyte to DNA damage requires further investigation. 

## 5. Conclusions 

PTEN/PI3K/AKT constitutes an important pathway regulating the signaling of multiple biological processes of female gametogenesis from PGC development to oocyte growth and folliculogenesis. In a number of cell types including PGCs as highlighted here, components of these pathways have been described as causal forces in tumors. Robust evidence sustains the notion that dysregulation of the activity of these components in PGCs can alter survival, proliferation, and differentiation of PGC, providing an important contribution to their transformation in tumorigenic cells. In addition, several data indicate that elements of these signaling pathways play essential roles in preserving the normal female fertility and reproductive lifespan. This provides a rationale for exploring the possible use of targeted therapies for the inhibition of the PI3K/PTEN/Akt and TSC/mTOR signaling cascades in certain ovarian dysfunctions including infertility and POF. Notably, the results of these studies represent the basis for improving current assisted reproductive methods including in vitro maturation of immature oocytes, and for the preservation of fertility by in vitro activation of follicles in cryopreserved ovarian tissue obtained from cancer patients or from women with reduced ovarian reserves.
